# Research on main influencing factors and complete support technology for dynamic pressure and large deformation roadway

**DOI:** 10.1038/s41598-023-31170-1

**Published:** 2023-03-13

**Authors:** Hai Rong, Kaipeng Guo, Dequan Sun, Mingkun Luo, Wei Dong, Bingjie Huo

**Affiliations:** 1 College of Mining, Liaoning Technical University, Fuxin, 123000 China; 2 Shandong Province Research Institute of Coal Geology Planning and Exploration, Jinan, 250104 China; 3 Engineering Laboratory of Deep Mine Rockburst Disaster Assessment, Jinan, 250104 China; 4 Zhangcun Coal Mine, Lu’an Environmental Protection Energy Development Co., Ltd., Changzhi, 046204 China; 5 College of Material Science and Engineering, Liaoning Technical University, Fuxin, 123000 China

**Keywords:** Civil engineering, Natural hazards, Environmental impact

## Abstract

To determine the main factors influencing dynamic pressure and large deformation roadways, a targeted set of support technologies was designed. The 2603 air inlet roadway of the Zhangcun coal mine in Lu'an, Shanxi Province, was taken as an example. The influence of the Wenwangshan South normal fault and in situ stress field on the dynamic pressure roadway was analyzed theoretically, and the main factors influencing this dynamic pressure and large deformation roadway under natural geological conditions were determined. The effect of the existing roadway support scheme was evaluated by field test methods such as nondestructive bolt testing. The influence of mining two working faces on the dynamic pressure and large deformation roadway was studied by the FLAC3D numerical simulation method. On this basis, a new grouting material was developed, a complete set of technical schemes of full-section integrated cooperative support of dynamic pressure and large deformation roadways was proposed, and the field application effect was verified. The results showed that under natural geological conditions, the 2603 air inlet roadway was located within the influence range of the Wenwangshan South normal fault, which was significantly affected and controlled by the fault. The included angle between the roadway extension direction and the maximum principal stress was 74°, which was not conducive to the stability of the roadway. The range of the roadway loose zone was large. Under the existing support conditions, the surrounding rock could not form a relatively stable structure, which was one of the main reasons for the large deformation of the surrounding rock in the dynamic pressure roadway. The 2603 air inlet roadway was affected by the mining of both the adjacent working face and the 2603 working face. The stresses were superimposed, and the roadway was greatly deformed and damaged. A new grouting material was developed. A crosslinking agent prepared by toluene diisocyanate and polyether polyol was added to the existing polyurethane material to form a new grouting material, and a complete supporting technical scheme was proposed. The field application results showed that the displacement and floor heave of both sides of the roadway were reduced by approximately 87%, the deformation and failure of the coal and rock mass of the roadway were effectively controlled, and the deformation of the dynamic pressure roadway was greatly reduced.

## Introduction

China’s coal resources were formed in many different periods, with complex maturity and distribution conditions. More than 90% of coal mine production originates from shaft mining^1^; thus, roadways play a vital role in coal mine production in China. The total length of coal mine roadways in China reaches 50,000 km, and most preparation roadways and recovery roadways are arranged in coal seams, of which the length of recovery roadways accounts for more than 60% of the total length of roadways^2^. Coal seam roadways will form dynamic pressure roadways under the influence of mining or other dynamic influences; the deformation of dynamic pressure roadways is often large and reworking work is frequent, which significantly increases the support cost, seriously affects the safe and efficient production of mines, and restricts the intensive production of coal mines. Therefore, the key to reducing the deformation of dynamic pressure roadways and the cost of support is to clarify the main factors affecting the large deformation of dynamic pressure roadways and to select and improve support measures in a targeted manner.

Regarding the main factors influencing the large deformation produced by dynamic pressure roadways, Li^3^ believed that the increase in roadway section area is one of the main factors of large deformation produced by dynamic pressure roadways. According to Liang^4^, the amount of roadway surface displacement increases faster at the early stage of roadway drivage. As the distance from the headway increases, the amount of roadway deformation gradually tends to stabilize. Zhang^5^ and Liu^6^ considered that when the coal seam retrieval roadway is located at a large burial depth, the mechanical properties of the top and bottom plates of the roadway will change, which is one of the main factors for the large deformation of the dynamic pressure roadway. Li^7^ considered that the main reason for the large deformation of dynamic pressure roadways is the stresses borne by the top plate of the roadway and the two gangs are released through the unsupported roadway bottom plate. Liu^8^ considered that the weak lithology of the top and bottom slabs of the roadway is the main factor influencing the large deformation and damage in the roadway. Sun^9^ considered that the control range of the anchor rod support structure is less than the damage depth of the roadway gang, and the failure of some anchor rods and anchor cables in the roadway gang is the main reason for the large deformation of the dynamic pressure roadway. Wang^10^ analyzed the phenomenon of damage of dynamic pressure roadways and the evolution law of surrounding rock stress and concluded that the mutual superposition of the mining-induced stress and vertical stress of coal columns is the main factor influencing large deformation of dynamic pressure roadways. Zheng^11^ studied the deformation and damage mechanism and surrounding rock reinforcement technology of a roadway group under strong dynamic pressure and large deformation and concluded that the stress concentration generated by dense coal pillars and workface retraction are the two main factors controlling the large deformation of the surrounding rock in the roadway group. Cai^12^ concluded that the main reason for the destruction of the surrounding rock in the roadway chamber is that the axial direction of the roadway is perpendicular or oblique to the direction of the maximum principal stress. Yuan^13^, based on the elastic‒plastic theory and field investigation and analysis, established the mechanical model of a circular roadway under a deep dynamic pressure environment, derived the implicit equation of the plastic zone boundary, and further revealed the large deformation mechanism of a deep dynamic pressure mining roadway. Kuai ^14^ used numerical simulation software such as FLAC3D and ANASYS and laboratory tests to optimize the parameters of support materials in accordance with the surrounding rock stress environment and roadway deformation characteristics, combined with the failure of roadway support materials in the failure section affected by dynamic pressure. Chen^15^ studied mechanical models of the microstructure of bolts, anchor cables and type I and anchor cables and sliding surfaces based on the deformation characteristics of dynamic pressure mining roadways in a fully mechanized caving face and the distributions of two types of sliding surfaces and proposed the support idea of “carrying the top and bottom, controlling the two sides”. Liu^16^, according to the stress state of the surrounding rock and geological conditions, determined that the transport crosscut roadway in the south wing of a mine should follow the principle of “rigid and flexible complementation, combination of long and short, timely initiative, coordination and control”. Wu^17^, under the influence of various factors, monitored the stress of the anchor cable and carried out a systematic stress analysis to give full play to the active support role of the anchor cable and provide basic data for the optimization of the support scheme. Wu^18^ proposed the control of the surrounding rock of the remaining roadway in a large mining height working face by using coupling and pressure equalizing support technology and achieved remarkable results.

This paper aims to study the influence of 2603 working face mining on the large deformation of a roadway, determine the factors influencing a dynamic pressure large deformation roadway in a wind tunnel, and then optimize the support parameters. On the basis of extensive research, we analyze the stress evolution and deformation of the roadway surrounding rock, select reasonable surrounding rock control technology, optimize the support mode and parameters of the back mining roadway in the Zhangcun coal mine, develop new grouting materials, and form a complete set of technologies for full-section integration and cooperative support of the dynamic pressure large deformation roadway in the Zhangcun mine. The research results will improve the safety and stability of the roadway, reduce the cost of support, and provide a reference for the roadway support of mines with similar conditions.

## Project background

**Figure 1:**
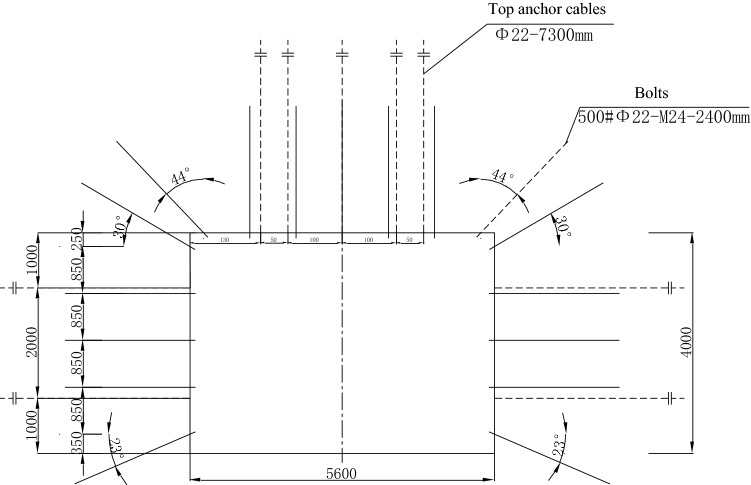
Support scheme of the air inlet roadway in 2603 working face.

**Figure 2:**
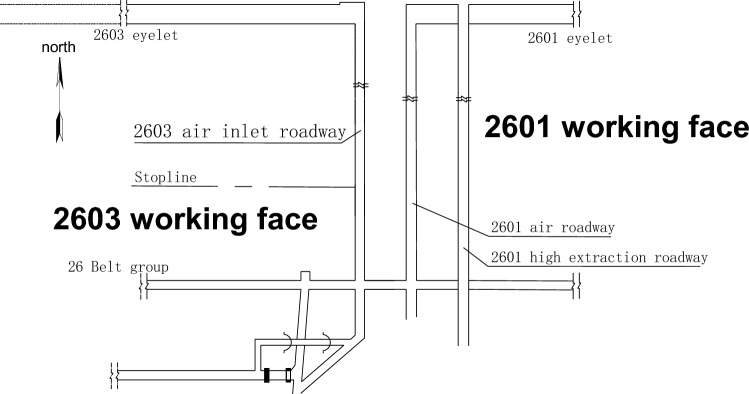
Layout of 2603 working face.

The Zhangcun coal mine mainly mines coal seam No. 3, which is a stable and mineable coal seam throughout the whole area, with an average thickness of 6.28 m. In the 26 mining area of the No. 3 coal seam, the 26 belt roadway group, 2603 air inlet lane and other lanes are seriously deformed by the repeated dynamic pressure from the working face, thus making them need to be repaired frequently. At the same time, the dynamic pressure also increases the maintenance cost of the lane and the difficulty of repairing the lane. Such roadways affected by mining or other dynamics form dynamic pressure roadways^19^. The 2603 air intake roadway is a typical dynamic pressure roadway in the 26 mining area, which adopts the joint support form of “anchor rod + anchor cable + steel ladder beam + W steel belt + metal mesh”, and the support form is shown in Fig. 1. Figure 2 illustrates the layout of the working face tunnel. The 2603 working face is located in the middle of the 26 mining area; the 26 mining area has an alley on its southern side, the minefield boundary (Wenwangshan South fault) is in the north, the 2601 working face mining void area is in the east, and the unmined area is in the west. The 2603 intake airway bears the mining-induced stresses due to 2601 working face mining and 2603 working face mining. During the mining period, the deformation of the tunnel surrounding rock is relatively violent: the total volume of deformation in the two sides is over 600 mm, the maximum sinkage of the roof plate is over 1100 mm, and the maximum bottom heave of the floor is over 3600 mm. Meanwhile, the anchor rod fractures from sporadically, and the complete roadway surrounding rock deforms irregularly.

## Analysis of the main factors influencing the dynamic pressure of a large deformation roadway under natural geological conditions

### Geological conditions

**Figure 3:**
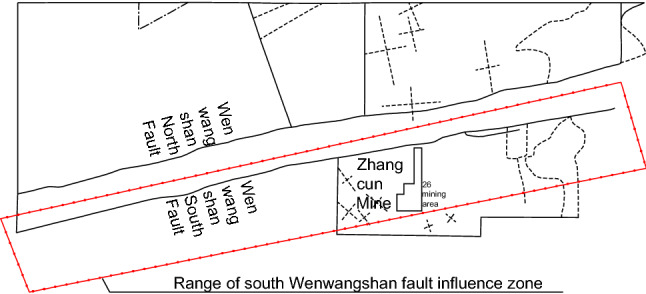
Relative position relationship between the Wenwangshan fault influencing area and the Zhangcun mine.

The Wenwangshan South normal fault extends eastward to Zhouwangshan, intersecting the Jinhuo fault zone (north by east 70°) and intersecting the Wenwangshan North fault (south by west 80°) when it extends westward to Hongshigou village. The relative positional relationship between the Wenwangshan fault influencing area and the Zhangcun mine is shown in Fig. 3.

According to the evaluation index of the fracture structure influence range in the evaluation index system of the geodynamic conditions^20^, the mine is affected by an active fracture when the linear distance between the minefield boundary and the fracture is less than the fracture influence range.

1 $$b = K.10h$$

where k is the activity coefficient (k = 1, 2, 3). When the fracture activity is strong, k = 3. When the fracture activity is moderate, k = 2. When the fracture activity is weak, k = 1. h is the vertical throw of the fracture (m).

According to the exploration results of the geological department, the Wenwangshan South normal fault has misplaced the Quaternary sediments and is a moderately active fault with an activity coefficient k of 2. The influence range of the Wenwangshan South normal fault at the northern boundary of the Zhangcun mine is 4.6–8.0 km, and all 26 mining areas are located within the influence range of the Wenwangshan South normal fault and thus are affected and controlled by it. The presence of the Wenwangshan fault increases the stress level in the area, which is not conducive to roadway support. At the same time, there are several small faults within the Zhangcun minefield, and the coal seams and rocks near the faults are broken such that sloughing and roof fall often occur, causing the bottom plate to be uneven, which affects the recovery operation and roadway support to a certain extent.

### Characteristics of the stress field

**Figure 4:**
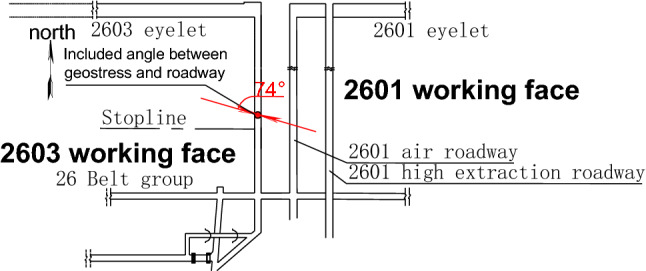
Included angle between the in situ stress direction and air inlet roadway of 2603 working face.

In May 2021, the Mining Design Division of Tiandi Technology Co Ltd. monitored the ground stress in the Zhangcun mine using the hydraulic fracturing method at three measurement points. Academician Hongpu Kang et al. concluded that the maximum horizontal principal stress direction was concentrated between N19.1° W and N72.9° W between the Wenwangshan South normal fault and the Egangshan North Main Fault^21^. Yan^22^ applied the integrated seismic source mechanism solution method and the grid search method, using fault data to obtain the characteristics of the orientation distribution of the tension axis at the junction of Jin, Ji and Yu. From the integrated seismic source mechanism solution method, it was determined that the maximum principal stress orientation in the Changzhi region is mainly oriented in the NW-WNW direction, and the dip direction is close to horizontal^23^. Therefore, the ground stress test results suggest the a maximum horizontal principal stress value of 16.79 MPa, a minimum horizontal principal stress value of 9.32 MPa, a vertical stress value of 13.01 MPa, and a maximum principal stress direction of N74.0°W, and we selected these data for analysis and calculation. The included angle between the in situ stress direction and the air inlet roadway of 2603 working face is shown in Fig. 4.

## Evaluation of the existing support program effect on the 2603 wind tunnel grouting anchor

### The test of the loose range

Before the excavation of the tunnel, the rock body bears the original rock stress and remains stable; after the excavation of the tunnel, the stress of the tunnel surrounding rock is redistributed, and a stress change zone and stress concentration appear in the surrounding rock. According to the theory of perimeter rock loosening circle support^24^, we use the perimeter rock structure detection method to measure the width and range of fissures in the 2603 inlet windway to determine the main deformation of the perimeter rock and the direction of the main fissures in the rock to accurately reveal the state of the underground engineering perimeter rock and the rock yield, the development of fissures, and the development of engineering perimeter rock deformation to speculate the range of the loosening circle.

According to the loosening circle test results, the roadway surrounding rock stability classification can be carried out. Table 1 shows the classification index of the coal seam roadway surrounding rock loosening circle.

**Table 1:** Classification index of the surrounding rock loose zone.

Surrounding rock classification	Category Name	Loose range of surrounding rock (mm)	Category Name	Support mechanism and method	Remarks
I	Komatsu moving ring	0–400	Stabilize the surrounding rock	Sprayed concrete support	Good integrity of the surrounding rock, unsupported
II	Middle loosening ring	400–1000	More stable surrounding rock	Anchor suspension theory, sprayed layer local support	There is a certain amount of damage to the rigid support
III		1000–1500	General surrounding rock	Anchor suspension theory, sprayed layer local support	There is a certain amount of damage to the rigid support
IV	Large loosening circle	1500–2000	General unstable surrounding rock (soft rock)	Anchor suspension theory, sprayed layer local support	Rigid support is damaged on a large scale, using retractable support
V		2000–3000	Unstable surrounding rock (softer surrounding rock)	Anchor rod combination with arch theory, spray anchor network support	There is a stabilization period for the surrounding rock deformation

We selected three test sections in the 2603 inlet airway to perform loose circle testing: at 645 m, 670 m and 700 m. Among them, 645 m is 18 m from the triangular coal, 670 m is the outer end of the overrun maintenance section, and 700 m is 30 m from the overrun section. The loose circle detection results show that the range of the loose circle of the 2603 wind tunnel roof is 2–4 m, and the range of the loose circle of the two sides is 6–7 m. The range of the loose circle of the 2603 wind tunnel roof is 2–4 m, which corresponds to the V–VI type of surrounding rock and the category of unstable to extremely unstable surrounding rock; the range of the loosening circle of the two sides is 6–7 m, which belongs to the VI type of surrounding rock and belongs to the category of extremely unstable surrounding rock. Considering the loose circle detection results, the current anchor support parameters used in the 2603 windway and other dynamic pressure lanes are as follows: top anchor spacing 0.85 m, row spacing 0.9 m, 7 rows each; gang anchor spacing 0.85 m, row spacing 0.9 m, 5 rows each; anchor type rebar anchor, diameter Ф22 mm, length 2400 mm; anchor cable support. The top anchor cable adopts a “three–two” arrangement: three anchor cables with a spacing of 1.5 m and row spacing of 1.8 m, and two anchor cables with a spacing of 2.0 m and row spacing of 1.8 m. The top anchor cable is steel strand anchor cable with a diameter Ф17.8 mm and length of 7300 mm; the two gang anchor cables have a spacing of 2.0 m and row spacing of 1.8 m and are steel strand anchor cables, with a diameter of Ф17.8 mm and length of 4300 mm. According to the loosening circle test results, the existing support parameters cannot meet the requirements of surrounding rock control, which is one of the main factors controlling the large deformation of dynamic pressure roadways.

### Pullout test

The purpose of the anchor pullout force test is to determine the anchorability of the roadway envelope and to evaluate the performance of the anchor, resin, and envelope anchoring system and the anchorage force of the anchor^25^. Three sets of pullout tests were carried out at 645–670 m in the 2603 inlet airway, and each set was pulled out three times. These tests were conducted to understand the performance of the anchoring system of the surrounding rock in the dynamic pressure and large deformation roadways of Zhangcun coal mine. The results of the anchor pullout test in the 2603 inlet airway of the Zhangcun coal mine are shown in Table 2.

**Table 2:** Bolt pullout test results of the 2603 air inlet roadway in the Zhangcun coal mine.

Test location	Grouping	Anchored section of the surrounding rock	Anchor type	Anchorage section length (cm)	Anchorage force average value (kN)	Failure form
2603 Intake Lane	Group 1	Muddy sandstone	φ22	120.8	181	Anchor section failure
				120.8	180	Anchor section failure
				120.8	184	Anchor section failure
	Group 2	Muddy sandstone	φ22	120.8	182	Anchor section failure
				120.8	182	Anchor section failure
				120.8	183	Anchor section failure
	Group 3	Muddy sandstone	φ22	120.8	182	Anchor section failure
				120.8	184	Anchor section failure
				120.8	182	Anchor section failure

The size of the anchorage force is related to the nature of the surrounding rock at the anchorage point and the anchorage effect. According to the regulations of the Lu’an Zhangcun coal mine, the anchor pullout force should meet the anchorage strength (120–190 kN) requirements. According to 3 sets of 9 pullout tests, the anchor section failed at an average anchor force of 182.2 kN when the anchor section length was 120.8 cm, which satisfied the anchor strength (120–190 kN) requirement.

This indicates that the anchor quality and anchor force are not the main factors influencing the large deformation of dynamic pressure lanes such as the 2603 inlet airway.

### Nondestructive testing of anchor rods

For the anchoring performance of anchor rods, nondestructive testing of anchor rods in the inlet airway of the 2603 working face was completed. Three locations were selected, in the same section as the peephole; 645 m, 670 m and 700 m.

The results of the nondestructive testing of the anchor rods showed that the integrity of the anchor rod support process was good and that the performance of the anchor rods was “excellent”. The anchor rod anchorage performance is not the main factor influencing the large deformation of dynamic pressure lanes such as the 2603 air intake lane.

In summary, under natural geological conditions, roadways such as the 2603 air intake roadway are seriously deformed due to the influence of ground stress, and according to the loosening circle test results, the existing support method is not sufficient to maintain roadway stability. In addition to the geological conditions, the roadway is affected by the mining of the working face, and further research is conducted on the influence of the mining of the working face on the roadway, such as the 2603 air intake roadway.

## Study on the effect of working face mining on the dynamic pressure of a large deformation roadway

**Figure 5:**
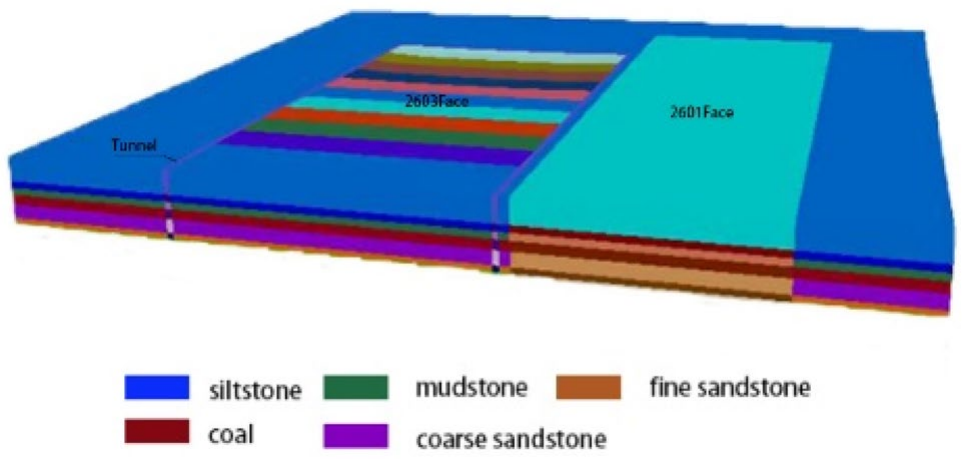
FLAC3D numerical model.

Detailed analysis of the impact of workface back mining on the 2603 air inlet lane, was performed via modeling and calculation of the 2603 workface as an example. The 2603 working face of the Zhangcun coal mine is located in the middle of the 26 mining area, with the 26 mining area alley to the south, the minefield boundary (Wenwangshan South Fault) to the north, the 2601 working face mining area to the east, and the unmined area to the west. The 2603 working face has a cut-hole length of 320 m, a recoverable length of 1153 m, and a recoverable reserve of 2.84 million tons. The 2603 working face uses the technique of leaving an alley along the roadway to assist in the working face mining. The working face 2603 uses the “Y + high extraction road” roadway layout. The finite difference software FLAC3D was used to establish a numerical simulation model based on the production and geological conditions of the 2601 and 2603 working faces and to analyze the surrounding rock stress distribution during working face mining through numerical calculation. The numerical model of working face mining was established as shown in Fig. 5.

The mechanical parameters of each coal seam are shown in Table 3. The boundary conditions of the computational model are determined as follows.

1. Constraints along the X-axis are imposed on the boundaries at both ends of the model X-axis, i.e., the boundary displacement in the X-direction is zero.
2. Constraints along the Z-axis are imposed on the boundary at both ends of the model Z-axis, i.e., the displacement of the boundary in the Z-direction is zero.
3. The model Y-axis (bottom) boundary imposes a fixed constraint along the Y-axis, i.e., the displacements in the Y-direction of the bottom boundary are all zero.
4. The top of the model is a free boundary.

**Table 3:** Physical and mechanical parameters of the coal seam, roof and floor in the working face.

Rock name	Density (kg m^−3^)	Tensile strength (MPa)	Bulk modulus (MPa)	Shear modulus (MPa)	Cohesion (MPa)	Internal friction angle (°)
Siltstone	2601	0.36	1700	1500	1.3	34
Coal	1267	0.1	100	100	0.2	21.53
Mudstone	2493	0.36	600	700	0.46	29.77
Coarse sandstone	2654	0.86	2100	1800	1.6	35
Fine sandstone	2594	0.66	1800	1600	1.4	33

Combining the results of ground stress measurements in the minefield, the model boundary load conditions were calculated as follows.

1. A stress of 16.94 MPa is applied in the X-axis direction of the model.
2. A stress of 9.32 MPa is applied in the Z-axis direction of the model.
3. A stress of 12.5 MPa is applied in the Y-axis direction of the model, an equivalent load of 12.5 MPa is applied to the upper part of the model, and the self-weight load is set in the Y-direction.

**Figure 6:**
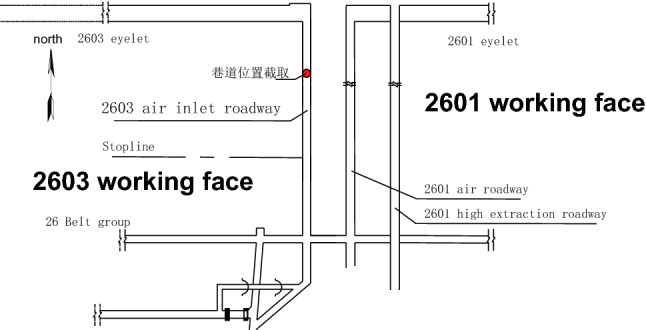
Location of the observation point in the roadway.

The wind tunnel selected observation point is located in the open cutting eye at 300 m, as shown in Fig. 6. The 2603 working face is to the right of the 2601 working face with or without the primary mining and secondary mining. Using FLAC3D numerical simulation of the 2603 roadway in the wind tunnel, the stress changes are studied, and the following describes the primary mining impact: when the 2601 working face advanced 50 m, the stress value of the bottom plate of the 2603 inlet tunnel was 10.83 MPa, the left gang was 19.72 MPa, and the right gang was 20.10 MPa; when the 2601 working face advanced 100 m, the stress value of the bottom plate of the 2603 inlet tunnel was 11.81 MPa, the left gang was 21.73 MPa, and the right gang was 23.59 MPa; when the 2601 working face advanced 150 m, the stress value of the bottom plate of the 2603 inlet tunnel was 12.91 MPa, and the left gang was 22.73 MPa. The bottom plate stress of the 2603 air-in tunnel is 12.91 MPa, the left gang is 22.73 MPa, and the right gang is 24.31 MPa; the bottom plate stress of the 2603 air-in tunnel is 13.20 MPa, the stress of the left gang is 24.08 MPa, and the stress of the right gang is 26.93 MPa when the 2601 working face is advanced 200 m; the bottom plate stress of the 2603 air-in tunnel is 16.59 MPa, the stress of the left gang is 27.52 MPa, and the stress of the right gang is 29.44 MPa when the 2601 working face is advanced 250 m. Regarding the impact of secondary mining, when the 2603 working face is not mined, the bottom plate stress of the roadway is 14.3 MPa, the stress of the left gang is 23.74 MPa, and the stress of the right gang is 25.24 MPa; when the working face is advanced 50 m, the bottom plate stress of the roadway is 15.9 MPa, the stress of the left gang is 24.40 MPa, and the stress of the right gang is 27.52 MPa; when the working face is advanced 100 m, the bottom plate stress of the roadway is 16.9 MPa, the stress of the left gang is 24.40 MPa, and the stress of the right gang is 27.52 MPa. When the workface is advanced 100 m, the stress of the roadway bottom plate is 16.9 MPa, the stress of the left gang is 25.72 MPa, and the stress of the right gang 30.50 MPa; when the workface is advanced 150 m, the stress of the roadway bottom plate is 18.9 MPa, the stress of the left gang is 27.72 MPa, and the stress of the right gang is 33.42 MPa; when the workface is advanced 200 m, the stress of the roadway bottom plate is 23.3 MPa, the stress of the left gang is 29.09 MPa, and the stress of the right gang is 36.22 MPa; when the workface is advanced 250 m, the bottom stress of the roadway is 26.03 MPa, the stress of the left gang 32.48 MPa, and the stress of the right gang is 39.73 MPa.

From the above analysis, it can be seen that the 2603 inlet roadway of the Zhangcun coal mine is affected by the mining-induced stresses of both the 2601 working face and 2603 working face. Under the influence of the primary mining of the 2601 working face, the stress concentration coefficient of the bottom plate of the 2603 inlet roadway is 1.68, and the stress concentration coefficient of both gangs is 1.47–1.72. Under the influence of the secondary mining of the 2603 working face, the stress concentration coefficient of the bottom plate of the 2603 inlet roadway is 2.64, and the stress concentration coefficient of both gangs is 1.74–2.32. The stress concentration coefficient of the 2603 inlet lane is 2.64, and the stress concentration coefficient of both gangs is 1.74–2.32. Compared with that after primary mining, the stress concentration coefficient of the 2603 inlet lane bottom plate is increased by 0.96, and the stress concentration coefficient of both gangs is increased by 0.27–0.60 under the influence of secondary mining of the 2601 and 2603 working faces. The change in the stress concentration coefficient of the 2603 inlet lane under the influence of mining is shown in Table 4. The stress concentration coefficient reflects the degree of stress concentration; the greater the stress concentration coefficient is, the greater the stress on the roadway^26^, and the superposition of the stress caused by back mining is the main reason for the large deformation and damage of the surrounding rock of the 2603 air intake lane.

**Table 4:** Change in the stress concentration coefficient of the 2603 air inlet roadway caused by mining.

Propulsion distance (m)	Primary mining-induced stress concentration coefficient			Secondary mining-induced stress concentration coefficient		
	Base plate	Left gang	Right gang	Base plate	Left gang	Right gang
0	1.00	1.00	1.00	1.45	1.27	1.48
50	1.09	1.05	1.18	1.61	1.30	1.61
100	1.20	1.16	1.38	1.72	1.37	1.78
150	1.31	1.21	1.42	1.91	1.48	1.95
200	1.34	1.29	1.57	2.37	1.55	2.12
250	1.68	1.47	1.72	2.64	1.74	2.32

## Formation and application of a complete set of technologies for integrated full-section collaborative support of dynamic pressure large deformation roadways

### Grouting anchor (cable) reinforcement program determination

According to the analysis results of factors such as the plastic destruction of the surrounding rock of the 2603 inlet airway, under natural geological conditions, ground stress has a great impact on the 2603 working face inlet airway, which is not conducive to maintaining the stability of the roadway, and the existing support method is not sufficient to maintain the stability of the roadway through the loosening circle test. Under the influence of mining, the bottom coal body undergoes plastic deformation first, which in turn leads to the stress of the two gangs and the overlying coal rock layer transferring to the bottom rock layer through the surrounding rock in the elastic‒plastic zone, causing damage to the 2603 air-in tunnel and the two gangs. The weakness of the self-supporting capacity of the 2603 air-in tunnel roadway is the root cause of its large deformation and damage, and improving and maintaining the self-supporting capacity of the surrounding rock is a reliable way to solve this kind of problem. For this situation, three kinds of grouting reinforcement plans were designed for the surrounding rock of the tunnel, and the specific implementation plans of the three grouting plans are shown in Table 5.

**Table 5:** Three grouting schemes.

Program serial number	Program name
Optimization plan I	Top plate and two gang anchor grouting
Optimization option two	Bottom plate anchor grouting
Optimization option three	Full-section grouting (top and gang anchor cable grouting and bottom slab anchor rod grouting)

The effect of the existing support and three grouting reinforcement optimization schemes are simulated and analyzed by FLAC3D numerical simulation, and the comparison of the effect of the existing support and three grouting optimization schemes is shown in Table 6.

**Table 6:** Comparison between existing support and three grouting optimization schemes.

Program serial number	Support solutions	Top slab sinking volume (mm)	Reduced comparison (%)	Base plate bottom drum volume (mm)	Reduced comparison (%)	The two gangs move closer to the volume (mm)	Reduced comparison (%)
Existing Program	Existing support	1120	–	3640	–	6050	–
Optimization scheme I	Grouting of the roof and both sides	970	13	3280	10	2090	65
Optimization option two	Bottom slab grouting	980	13	1890	48	2620	57
Optimization scheme three	Full-section grouting	630	44	1210	67	1570	74

According to the simulation results, under the full-section grouting condition, the top slab sinkage is 630 mm, which is 44% lower than the original support, the bottom slab bottom bulge is 1210 mm, which is 67% lower than the original support, and the two supports of the roadway are shifted closer by 1570 mm, which is 74% lower than the original support. This result shows that the effect of full-section grouting is the best with optimization option three. The full-section grouting support scheme has the best effect on the control of the roadway surrounding rock compared with the anchor cable grouting of the two supports and the anchor rod grouting of the bottom plate; therefore, the full-section grouting support scheme is recommended for roadway surrounding rock reinforcement.

## Research and development of new grouting materials

At present, the material selected for roadway grouting in the Zhangcun coal mine is polyurethane material. To meet the requirements of the accounting culture, the performance of the support material is further improved on the premise of reducing the existing cost by 5%. To this end, new grouting materials were developed.

1. Research process

The materials and reagents used in the experiments are shown in Table 7, where isocyanate and polyether polyol are the raw materials for the esterification reaction, and organotin is used as a catalyst to control the rate of the gelation and foaming reactions. The experiments focused on the effect of the cross-linking agent dosage on the compression properties and bonding performance of the reinforcing material system. The crosslinker was formulated with toluene diisocyanate and polyether polyol. The amount of cross-linking agent was added at 0, 2%, 4%, 6%, 8%, 10% and 12% of the coagulation force polyurethane A component.

**Table 7:** Materials and reagents used in the experiment.

Serial number	Name	Source	Purity	Molecular formula	English name
1	Toluene diisocyanate	Langfang Chenhao Chemical Co	–	CHNO	Isocyanic acid
2	Polyether polyol	Nantong Qianhe Chemical Co	–	–	Polyether polyol
3	Tin-based catalysts	Shanghai Aladdin Biochemical Technology Co	95%	Organotin	Dibutyltin dilaurate
4	Crosslinker	Qingdao Lianmei Chemical Co	–	–	Mold releaser for polyurethane

**Figure 7:**
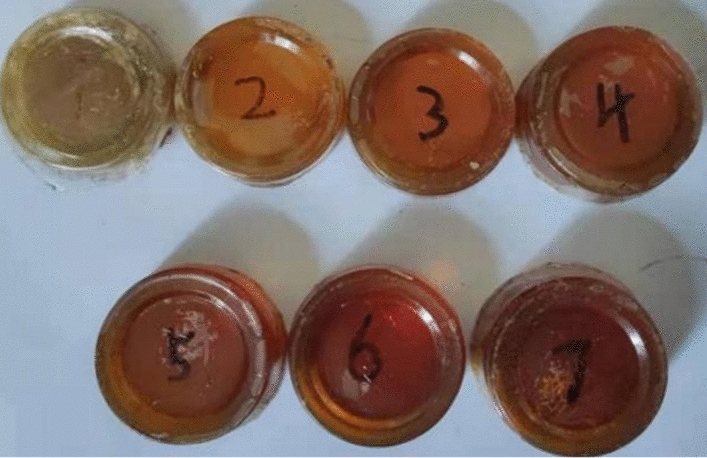
Compressed specimen.

The two-component polyurethane reinforcement was prepared, poured into uniform-size cylindrical plastic paper cups before the drug started to set, and demolded after it had completely set and formed. The seven groups of demolded samples were sanded with sandpaper one by one to a cylinder 30 mm in height and 50 mm in diameter and left for compression testing. The compression specimens are shown in Fig. 7.

**Figure 8:**
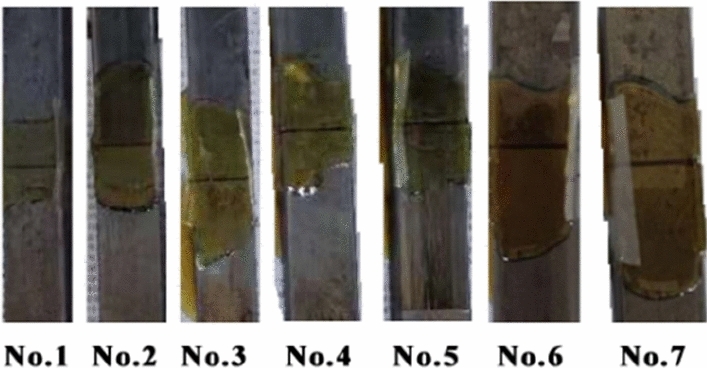
Bonding sample.

Fourteen stainless steel strip pieces with a size of 100 mm × 25 mm × 15 mm were prepared and wiped with anhydrous ethanol, and the surfaces of the samples were thoroughly cleaned. In pairs, a 25 mm × 15 mm plane butt joint was created with a 1 mm gap. Then, the 7 groups of test pieces were placed on the horizontal table. Nest, the following steps were taken: Prepare the two-component polyurethane reinforcing agent. Before it starts to solidify, use a pipette to absorb a proper amount of medicine, fully inject it into the gap reserved for the butt joint sample, and let it stand for more than 24 h to make it fully and completely solidify, as shown in Fig. 8, and leave it for tensile testing.

(B) Research results

**Figure 9:**
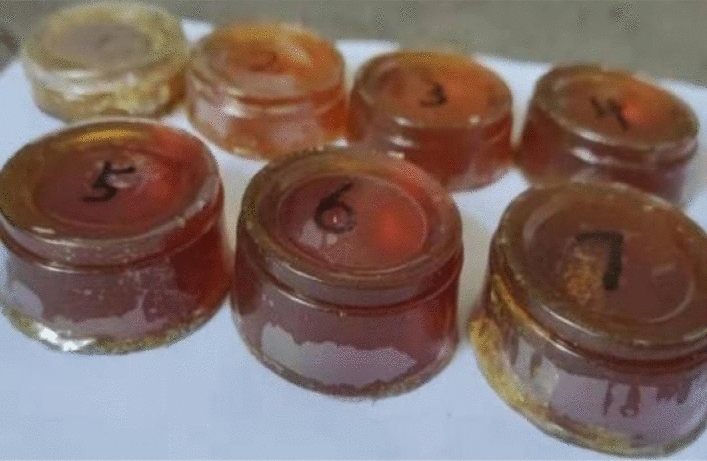
Compressed specimens.

**Figure 10:**
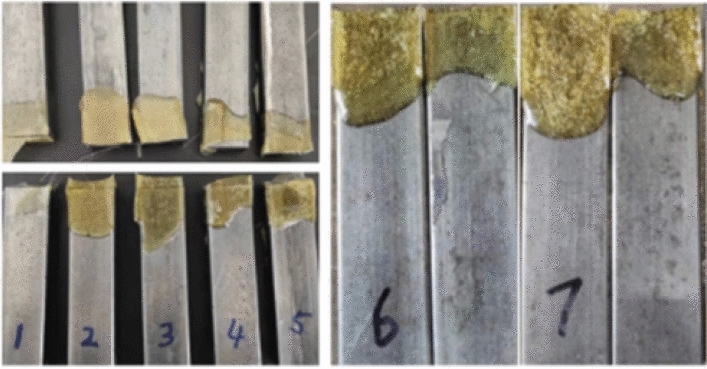
Adhesion test sample after tensile testing.

Each group of specimens produces a slight difference in deformation variables, and the specimens after the compression experimentation are shown in Fig. 9. The samples after the tensile testing are shown in Fig. 10.

**Figure 11:**
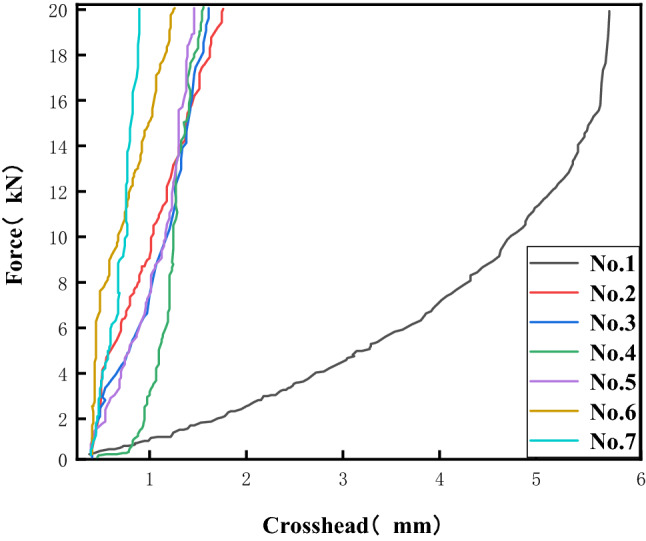
Compression test curve.

The force-compaction curves of the seven sets of compressed specimens drawn by using origin software are shown in Fig. 11. Under the condition of loading up to 20 kN, the curves follow the general pattern of gradually decreasing in compaction from No. 1 to No. 7, which shows that the strength of the specimens gradually increases.

**Figure 12:**
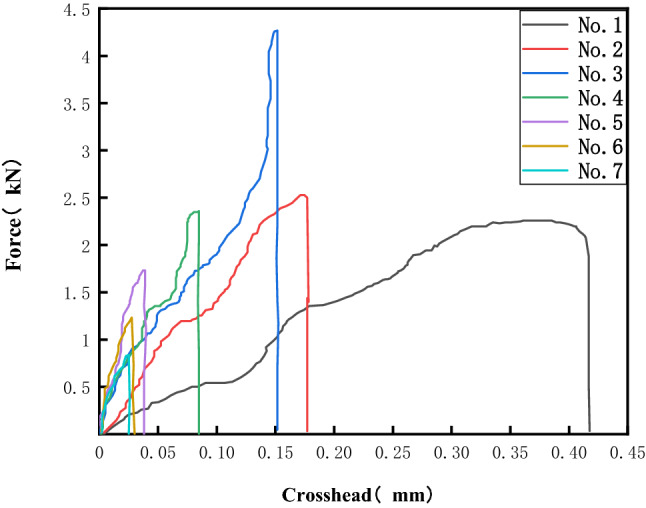
Force elongation curve.

It can be concluded that in the raw material, the cross-linking agent content and the hardness of polyurethane reinforcement are positively correlated and that the higher the cross-linking agent content is, the higher the strength of polyurethane reinforcement. The force–elongation curves of the seven groups of bonded specimens drawn by using Origin software are shown in Fig. 12. Under the condition of gradual loading, the curve roughly tends to decrease from No. 1 to No. 7 elongation as a whole, and the load applied at the time of fracture first increases and then decreases. There is a relationship between the bonding performance of the specimen and the ratio of raw materials.

When the cross-linking agent combined with toluene diisocyanate and polyether polyol was added, the bond strength of sample No. 3, i.e., with 4% cross-linking agent, was the largest and 1.87 times that without the cross-linking agent; the elastic modulus of sample No. 7, i.e., with 12% cross-linking agent, was the largest and 6.33 times that without the cross-linking agent. Considered together, the elastic modulus is 3.56 times that without the cross-linking agent, and the bond strength is 1.87 times that without the cross-linking agent when 4% cross-linking agent is added to the existing support material.

### Field applications

**Figure 13:**
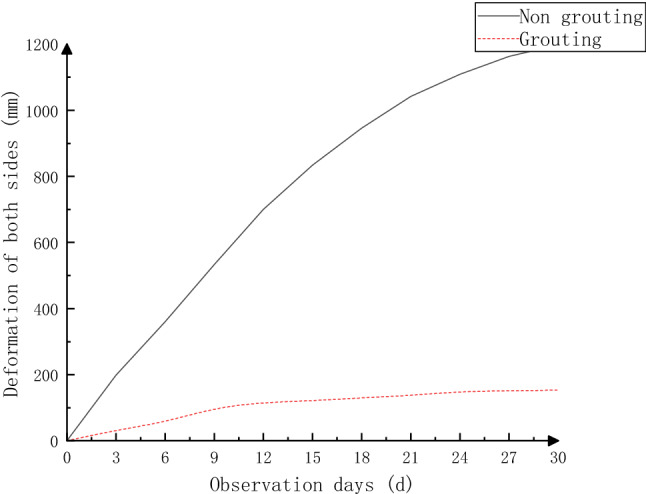
Comparison of deformation between the nongrouted and grouted sides.

**Figure 14:**
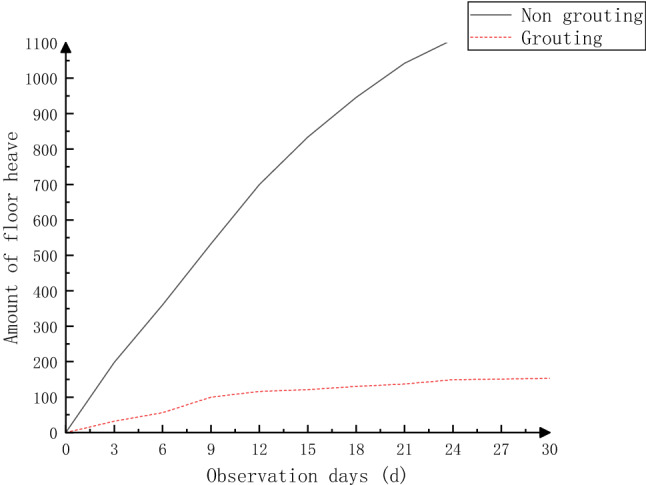
Comparison of nongrouting and grouting bottom heave results.

To study the field application effect of the new grouting material in the full-section grouting method, 510–530 m of the 2603 air-inlet roadway was selected as the test roadway, and the technology was applied in the field. To verify the reinforcement effect after grouting, two monitoring sections were set up at 520 m (grouted section) and 490 m (nongrouted section), and the crisscrossing method was used to monitor and compare the displacement of both sides of the roadway and the displacement of the top and bottom plates. The comparison of the deformations and bottom bulges of the nongrouted section and grouted section are shown in Figs. 13, 14.

Through comparative analysis, during the 30-day monitoring period, the bottom drum volume of the nongrouted section reached 1198 mm, and the displacement of both gangs reached 1043 mm. The bottom drum volume of the roadway changed considerably at the beginning, but the increase in the bottom drum volume decreased at the later stage, and the displacement of both gangs increased almost linearly. After grouting, the bottom drum volume and the displacement of both gangs were controlled, the maximum bottom drum volume was 130 mm, the maximum displacement of both gangs was 153 mm, and the displacement and bottom drum volume of both gangs were reduced by approximately 87%. These findings show that the original broken coal rock body was solidified into a complete whole under the action of slurry after the roadway grouting reinforcement, so the anchor rods and anchor cables were fairly effective. After adding the new material, the deformation volume was further reduced by 13% compared with the optimized results of the numerical simulation, and the effect of the new grouting material was more significant, which effectively controlled the deformation and damage of the coal rock body of the roadway, indicating that the stability of the roadway was improved after grouting reinforcement.

## Conclusion

1. Zhangcun coal mine 2603 wind tunnel is located near the Wenwangshan Mountain South normal fault and is influenced by it; the tunnel is arranged along the north‒south direction, and the maximum principal stress orientation is 74°. The ground stress has a great impact on the 2603 working face in the wind tunnel, which is not conducive to maintaining the stability of the tunnel.
2. Anchor quality, anchor force and anchor performance are not the main factors influencing the large deformation of dynamic pressure roadways such as the 2603 air intake roadway; the large loosening circle and the inability of the surrounding rock to form a relatively stable structure under the existing support conditions are some of the main reasons for the large deformation of the surrounding rock in the studied dynamic pressure roadway.
3. The 2603 wind tunnel is affected by the mining-induced stresses of the 2601 working face mining and 2603 working face mining, the stress concentration coefficient of the 2603 wind tunnel bottom plate is 2.64, and the two support stress concentration coefficients are 1.74–2.32. The superposition of stress caused by back mining is the main reason for the large deformation and damage of the 2603 wind tunnel surrounding rock.
4. According to the main factors influencing the large deformation and damage of the dynamic pressure lane, such as the 2603 air intake lane, a new support method of full-section grouting was determined. The specific parameters are as follows: top and bottom plates adopt φ22 × 2400 mm rebar anchor rods, with interrow spacing of 850 × 1000 mm and 7 sets in a row; top anchor rods adopt φ17.8 × 7300 mm grouted anchor rods, with a spacing of 1500 × 2000 mm and 2 sets in each row; the left and right gangs adopt φ22 × 2400 mm threaded steel anchor rods, and the distance between rows of anchor rods is 800 × 1000 mm, with 5 sets in a row; the anchor cables adopt φ17.8 × 4300 mm grouted anchor cables, and the distance between anchor cables is 1200 × 2000 mm, with 2 sets in each row.
5. A new grouting material was developed with the addition of a cross-linking agent formulated from toluene diisocyanate and polyether polyol to the existing coagulation force polyurethane. The bond strength was the highest at 4% cross-linking agent, which was 1.87 times that without cross-linking agent, and the elastic modulus was the highest at 12% cross-linking agent. A field test was carried out at 520 m in the 2603 wind tunnel, and the result was that the deformation of the tunnel surrounding rock was significantly reduced and the effect was remarkable.

## Data Availability

The data used to support the findings of this study are available from the corresponding author upon request.
